# Lack of association between endothelial nitric oxide synthase (NOS3) gene polymorphisms and suicide attempts

**DOI:** 10.1186/1744-9081-3-32

**Published:** 2007-07-02

**Authors:** Pilar A Sáiz, Maria P García-Portilla, Begoña Paredes, Celso Arango, Blanca Morales, Victoria Alvarez, Eliecer Coto E, Teresa Bascarán, Manuel Bousoño, Julio Bobes

**Affiliations:** 1 Department of Psychiatry, School of Medicine, University of Oviedo, Julián Clavería 6 – 3°, 33006, Oviedo, Spain; 2 Emergency Room, San Agustin Hospital, Camino de Heros 4, 33400, Avilés, Spain; 3 Department of Psychiatry, Hospital General Universitario Gregorio Marañón, Ibiza 43, 28009, Madrid, Spain; 4 Laboratory of Molecular Genetics, Central University Hospital of Asturias, Celestino Villamil sn, 33006, Oviedo, Spain

## Abstract

**Objective:**

The aim of this study is to investigate the association between two polymorphisms of endothelial nitric oxide synthase (NOS3) and suicide attempts.

**Methods:**

We genotyped 186 suicide attempters and 420 unrelated healthy controls. The following polymorphisms were analysed: T-786C and 27-bp repeat in intron 4.

**Results:**

No significant differences were found in genotype or in allelic distribution of the aforesaid polymorphisms. There were also no differences in the genotype distribution or allelic frequencies when separately assessing males and females or impulsive and non-impulsive attempters and normal controls. Estimated haplotype frequencies were similar in both groups.

**Conclusion:**

Our data do not support the hypothesis that genetically determined changes in the NOS3 gene confer increased susceptibility for suicidal behavior.

## Findings

There is good evidence, from both epidemiological and genetic association studies, that suicidal behavior, a serious world-wide problem, is familially transmitted, and that a substantial proportion of the variation in liability is explained by genes (approximately 45% heritability) [1,2].

Nitric oxide (NO) is produced from L-arginine by the action of the nitric oxide synthase (NOS). There are three NOS isoforms (NOS1, NOS2, and NOS3). NOS3 (also called endothelial NOS, or eNOS) is the predominant isoform in most tissues [3]. NOS3 is involved in the proliferation of neuronal progenitor cells, which might be implicated in the pathophysiology of depressive disorders [4,5]. Prior genetic association studies have suggested a possible role of NOS3 gene polymorphisms in affective disorders [5] and suicidal behavior [6].

The NOS3 gene is located on chromosome 7q35.1. There are several NOS3 polymorphisms and it has been suggested that at least two of these (a promoter single nucleotide polymorphism (SNP), T-786C (rs2070744), and a 27-bp repeat in intron 4) result in a reduction in NOS3 gene promoter activity [7] and function as an epistatic enhancer element depending on T-786C genotype [8], respectively.

In this study, we hypothesized that two functional genetic variants of the NOS3 gene (T-786C and 27-bp repeat in intron 4 would confer susceptibility to suicidal behavior.

One hundred and eighty-six consecutive suicide attempters (SA) admitted to the emergency room at San Agustin Hospital in Asturias, Northern Spain (mean age (SD) = 35.00 (11.9) years, 36.6% males) were enrolled in the study. Attempted suicide was defined according to the proposed WHO definition [9]. SA were assessed during the first 24 hours after the attempt using the Suicidal Intent Scale (SIS) [10]. Most of suicide attempts (88.7%) were non-violent (i.e., overdose, poisoning, or gas).

The control group consisted of 420 unselected healthy individuals. DSM-IV criteria derived from the Mini-International Neuropsychiatric Interview (MINI), were used to rule out any psychiatric disorder among normal controls. The mean age (SD) was 40.6 (11.3) years, and 51.4% were males.

To minimize the problem of "population stratification," all individuals were of Caucasian Spanish origin, shared similar sociodemographic profiles, and were comparable with respect to the geographic origin of their families.

The study was conducted according to the provisions of the World Medical Association Declaration of Helsinki, ethical approval of the study was granted by the local Institutional Review Board (IRB), and all participants gave their informed consent.

Genotyping was performed blind to clinical data and in duplicate, with patients and controls analyzed side by side to eliminate errors in genotyping. NOS3 gene polymorphisms were identified according to previously published methods [11].

Differences between allele and genotype frequencies were assessed using a chi-square test, and p ≤ 0.05 was considered significant. Odds ratios (ORs) and their Cornfield's confidence intervals (95% CIs) were also calculated. Suicide attempts were divided into two subgroups: impulsive and non-impulsive attempts, following the definition of attempt planning proposed by Díaz et al. [12] using the SIS (in 13 subjects, it was impossible to administer the SIS due to clinical condition). In accordance with the definition used, a score of 6 on the planning subscale was used to classify attempts as impulsive (n = 111 (64.2%)) or non-impulsive (n = 62 (35.8%)). Most of the impulsive attempts (n = 103 (92.8%)) were non-violent. Pairwise linkage disequilibrium (LD) and haplotype analyses were also performed.

The total number of samples analysed differed between SNPs because of sample exhaustion or repeated assay failure. Some degree of LD was found, independent of disease, between NOS3 T-786C and NOS3 VNTR polymorphisms (R value (Cramer'sV) = 0.252, p < 0.00001), but the LD was not complete enough to warrant not genotyping both polymorphisms.

The distribution of the genotypes and allelic frequencies for the NOS3 T-786C and NOS3 27-bp repeat in intron 4 polymorphisms in SA and controls is summarized in Table 1. No significant difference was observed in the genotype distribution or in allelic frequencies between the unrelated SA and controls, in the studied polymorphisms. The whole sample is large enough to detect a relative risk of 2 with a statistical power of 85% and significance of 5% (two-tailed test), assuming a minimum allele frequency of 14% in the healthy Caucasian population [11]. There were also no differences in the genotype distribution or allelic frequencies when separately assessing males and females or impulsive and non-impulsive attempters and normal controls (data not shown). The minimum statistical power when analyzing subgroups of samples was 75% to detect a relative risk of 2.5.

**Table 1:** Polymorphisms of NOS3 in suicide attempters and controls

	NOS3 T-786C	NOS3 27-bp, intron 4
Genotype		
Suicide attempters [n (%)]	53 (28.8%) TT	6 (3.2%) 44
	92 (50.0%) CT	48 (26.0%) 45
	39 (21.2%) CC	131 (70.8%) 55
		
Control frequencies [n (%)]	153 (36.4%) TT	23 (5.5%) 44
	197 (46.9%) CT	105 (25.0%) 45
	70 (16.7%) CC	291 (69.5%) 55
		
chi-square (df)	3.89 (2)	1.42 (2)
p value	0.143	0.491

Alleles		
Suicide attempters [n (%)]	198 (0.54) T	60 (0.16) 4
	170 (0.46) C	310 (0.84) 5
		
Control frequencies [n (%)]	503 (0.60) T	151 (0.18) 4
	337 (0.40) C	687 (0.82) 5
		
chi-square (df)	3.88 (1)	0.579 (1)
p value	0.049	0.447
OR (95% CI)	0.78 (0.61–0.99)	0.88 (0.63–1.22)

NOS3 = nitric oxide synthase; bp = base pairs; df = degrees of freedom; OR = Odds Ratio; CI = Confidence Interval

A total of 4 haplotypes were estimated by the Genecounting program to have non-zero frequencies. No significant differences were found in the frequencies of these haplotypes between SA and controls: likelihood ratio test (LRT) = 5.100, df = 3, p = 0.165.

No association between NOS3 gene polymorphisms and suicidal behavior was found in our sample. However, a marginal association was found between the NOS3 -786C allele and suicidal behavior (this association would disappear if a Bonferroni correction for multiple comparisons were applied). Our healthy control group shows genotypic and allelic frequencies similar to those previously found in other samples of Caucasian healthy controls [5,11,13,14].

Our results agree, at least in part, with the only prior report [6] looking for a possible association between NOS3 gene polymorphisms and suicidal behavior. The single marker analysis of their sample showed a protective effect of the rs891512 A-allele (p = 0.007), but no association with the NOS3 T-786C polymorphism. However, they found a protective haplotype C-T-A (T-786C/rs1799983, also called Glu298Asp/rs891512) (p = 0.01) with a pronounced effect against suicide completion (p = 0.005). On the other hand, there are no prior reports searching for an association between the 27-bp repeat polymorphism in intron 4 and suicidal behavior.

Type I error was minimized in two ways: i) our study has sufficient statistical power to prove an epidemiologically relevant impact of hereditary variations in the studied gene, ii) both patients and controls were matched for ethnicity and drawn from a homogeneous population. A possible limitation is that different psychiatric diagnoses are included in the SA sample. However, we agree with a prior report that suggests that liability to suicidal behavior might be familially transmitted as a trait independent of Axis I and II disorders [15,16].

In conclusion, the results of our study do not support the role of NOS3 gene polymorphisms in suicidal behavior.

## Competing interests

The author(s) declare that they have no competing interests.

## Authors' contributions

PAS designed the study, analyzed the data, contributed to data interpretation, and was the principal author of the manuscript. BP and TB carried out the data collection and contributed to data interpretation and manuscript preparation. BM, VA, EC, genotyped all participants and contributed to data interpretation and participated in manuscript preparation. MPGP, CA, MB, and JB contributed to the conception of the study, data interpretation, and manuscript preparation. All authors have read and approved this manuscript.

## References

[B1] Arango V, Huang YY, Underwood MD, Mann JJ (2003). Genetics of the serotonergic system in suicidal behavior. J Psychiatr Res.

[B2] Courtet P, Jollant F, Castelnau D, Buresi C, Malafosse A (2005). Suicidal behavior: relationship between phenotype and serotonergic genotype. Am J Med Genet C Semin Med Genet.

[B3] Brown GC (1999). Nitric oxide and mitochondrial respiration. Biochim Biophys Acta.

[B4] Reif A, Schmitt A, Fritzen S, Chourbaji S, Bartsch C, Urani A, Wycislo M, Mossner R, Sommer C, Gass P, Lesch KP (2004). Differential effect of endothelial nitric oxide synthase (NOS-III) on the regulation of adult neurogenesis and behaviour. Eur J Neurosci.

[B5] Reif A, Strobel A, Jacob CP, Herterich S, Freitag CM, Topner T, Mossner R, Fritzen S, Schmitt A, Lesch KP (2005). A NOS-III haplotype that includes functional polymorphisms is associated with bipolar disorder. Int J Neuropsychopharmacol.

[B6] Mandelli L, Rujescu D, Giegling I, Schneider B, Hartmann AM, Schnabel A, Maurer K, Möller HJ, De Ronchi D, Serretti A (2006). HTR2C, HTR1A, NOS-I and -III gene variants in suicide attempters and completers. Am J Med Genet B Neuropsychiatr Genet.

[B7] Nakayama M, Yasue H, Yoshimura M, Shimasaki Y, Kugiyama K, Ogawa H, Motoyama T, Saito Y, Ogawa Y, Miyamoto Y, Nakao K (1999). T-786-C mutation in the 5'-flanking region of the endothelial nitric oxide synthase gene is associated with coronary spasm. Circulation.

[B8] Wang J, Dudley D, Wang XL (2002). Haplotype-specific effects on endothelial NO synthase promoter efficiency: modifiable by cigarette smoking. Arterioscler Thromb Vasc Biol.

[B9] De Leo D, Burgis S, Bertolote JM, Kerkhof A, Bille-Brahe U, de Leo D, Bille-Brahe U, Kerkhof JF, Schmidtke A (2004). Suicidal behaviour among young people. Suicidal Behaviour Theories and Research Findings.

[B10] Beck AT, Schuyler D, Herman I, Beck AT, Resnick HLP, Lettieri DJ (1974). Development of suicidal intent scales. The prediction of suicide.

[B11] Asensi V, Montes AH, Valle E, Ocana MG, Astudillo A, Alvarez V, Lopez-Anglada E, Solis A, Coto E, Meana A, Gonzalez P, Carton JA, Paz J, Fierer J, Celada A (2007). The NOS3 (27-bp repeat, intron 4) polymorphism is associated with susceptibility to osteomyelitis. Nitric Oxide.

[B12] Diaz FJ, Baca-Garcia E, Diaz-Sastre C, Garcia Resa E, Blasco H, Braquehais Conesa D, Saiz-Ruiz J, de Leon J (2003). Dimensions of suicidal behavior according to patient reports. Eur Arch Psychiatry Clin Neurosci.

[B13] Hassan A, Gormley K, O'Sullivan M, Knight J, Sham P, Vallance P, Bamford J, Markus H (2004). Endothelial nitric oxide gene haplotypes and risk of cerebral small-vessel disease. Stroke.

[B14] Tanus-Santos JE, Desai M, Flockhart DA (2001). Effects of ethnicity on the distribution of clinically relevant endothelial nitric oxide variants. Pharmacogenetics.

[B15] Brent DA, Bridge J, Johnson BA, Connolly J (1996). Suicidal behavior runs in families. A controlled family study of adolescent suicide victims. Arch Gen Psychiatry.

[B16] McGuffin P, Marusic A, Farmer A (2001). What can psychiatric genetics offer suicidology?. Crisis.

